# The national burden of scabies in Germany: a population-based approach using Internet search engine data

**DOI:** 10.1007/s15010-022-01763-5

**Published:** 2022-02-08

**Authors:** Jing Wu, Linda Tizek, Melvin Rueth, Hannah Wecker, Alphina Kain, Tilo Biedermann, Alexander Zink

**Affiliations:** Department of Dermatology and Allergy, School of Medicine, Technical University of Munich, Munich, Germany

**Keywords:** Scabies, Google analysis, Dermatology, Scabies therapy

## Abstract

**Purpose:**

Scabies is a World Health Organization-defined neglected tropical disease and a growing public health issue worldwide. It is difficult to obtain reliable data on prevalence due to the lack of standardized tests. The aim of this study was to assess scabies online search behavior in Germany to identify local differences using Google search volume.

**Methods:**

Google Ads Keyword Planner was used to investigate the scabies-related search volume for Germany as a whole, its 16 federal states, and 15 large cities for the period from January 2016 to December 2019. The identified search terms were qualitatively categorized and critically analyzed.

**Results:**

A total of 572 keywords with an overall search volume of 11,414,180 searches regarding scabies were identified in Germany. The number of searches was higher in winter than in summer, with a national peak in March 2018. Around 30.6% of the searches regarding scabies therapy (*n* = 978,420) were related to home remedies. Regarding body localization, most searches focused on the whole body (*n* = 109,050), followed by head (*n* = 89,360) and the genital area (*n* = 28,640).

**Conclusions:**

The analysis of Google search data provides an overview of the populations’ interest regarding scabies. The analysis can detect local peaks and assess the relevance of scabies at individual localizations of the body. The study highlighted current possible shortcomings in the therapy of scabies. It also underlined the importance of improving awareness regarding scabies so that affected individuals can consult a doctor earlier for treatment.

**Supplementary Information:**

The online version contains supplementary material available at 10.1007/s15010-022-01763-5.

## Introduction

Scabies is a common infectious skin disease caused by the mite *Sarcoptes scabiei* var.* hominis* [1]. More than 200 million people are affected by scabies worldwide, with people living in tropical areas being especially affected by the disease [2]. For example, the prevalence is reported to be as high as 71.4% in Papua New Guinea [3].

Scabies significantly reduces the quality of life due to itch [2] and is responsible for 0.21% of disability-adjusted life years (DALYs, the unit of measure for loss of health life-years) worldwide [2, 4]. To emphasize the international relevance of scabies for public health care and to draw attention to the need for global cooperation in controlling the disease, the World Health Organization (WHO) classified scabies as a ‘neglected tropical disease’ in 2017 [4]. Scabies is also characterized by many frequently underestimated complications and secondary manifestations. Constant scratching of the skin creates a defective skin barrier, which in turn serves as an entry point for bacteria (especially Streptococcus pyogenes and Staphylococcus aureus) and can favor superinfections [5]. Bacterial infections can in turn lead to impetigo and corresponding secondary diseases such as rheumatic fever, glomerulonephritis, and sepsis [6].

Scabies does also occur in regions of temperate climate. Scabies prevalence has increased clearly in recent years in Germany [7]. Between 2014 and 2016, the number of documented cases of treated scabies in parts of Germany increased by around 200% from 6579 to 19,560 cases [8]. In addition, the prescription of antiscabiosa drugs among insured people of a large German health insurance company increased by 60% from 2016 to 2017 [9]. Although these studies indicated an increase in scabies, they did not provide a comprehensive overview of the scabies prevalence in Germany, as they only included affected people who sought medical help.

A method to represent the medical need associated with a certain disease is the analysis of Internet search volume [10]. Previous studies on pruritus [11], skin cancer [12], and borreliosis [13] demonstrated that Google data are suitable to reflect the interest of the German population in various diseases since Google is used by over 95% of Germans as an online search engine [14]. Additionally, it was demonstrated that the incidence and mortality of various tumors in the United States (US) correlated with the associated Google search volume [15, 16] and that confirmed Middle East Respiratory Syndrome cases in Korea could be traced using Google search data [16]. These studies indicated that Internet search data can support epidemiological surveillance of a disease to some extent, although it should be considered that mass media coverage or a general higher health-related search behavior can also increase the number of searches regarding diseases [17].

As the share of Internet users in Germany was 86% in 2019, with an upward trend [18], Internet search engine analyses are a beneficial method to represent the interest of the Google users in Germany regarding certain diseases. Thus, the aim of this study was to perform a comprehensive assessment of the search behavior related to scabies in Germany as a whole, in its federal states, and in some selected cities to identify geographical differences in search frequency and interest.

## Methods

### Study design

A four-year retrospective study examining the search volume regarding scabies was performed by analyzing the data provided by the Google Ads Keyword Planner. Google Ads Keyword Planner is primarily used for marketing strategies, but as the tool provides the average monthly search volume for identified keywords, the tool can also be used to answer scientific questions [11, 12, 19]. To identify the search volume for a specific topic, chosen keywords, which were ‘scabies’ and the German lay word ‘Krätze” in this study, are entered in the tool, which then provides a list of the most relevant related search phrases. For each identified keyword the absolute number of searches is provided for the last 48 months and thus, the search volume between January 2016 and December 2019 was investigated in this study. In addition, the region and the language for which the data is to be provided must be selected. In this study, only users in Germany with a German Internet protocol whose language preference was German were included in the study. In addition to the nationwide search volume, this study also examined the search volume across the 13 German federal states, the 3 city-states (Berlin, Bremen, and Hamburg), and 15 selected cities across Germany (Dortmund, Dresden, Frankfurt, Freiburg, Hannover, Kiel, Cologne, Leipzig, Magdeburg, Munich, Muenster, Nuremberg, Rostock, Saarbrucken, and Stuttgart).

As this study used generally available and anonymized data that do not allow any conclusions to be drawn about individuals, the consent of the Google customers and approval by an accredited safety committee was not required for this study.

### Classification of search terms

All keywords were analyzed qualitatively. A total of 15 categories were identified to which the keywords were assigned: (1) general (e.g., ‘scabies illness’), (2) therapy (e.g., ‘lavender oil for scabies’), (3) localization (e.g., ‘scabies genital area’), (4) mode of infection (e.g., ‘scabies transmission’), (5) children (e.g., ‘scabies in infants’), (6) animals (e.g., ‘scabies cats’), (7) symptoms (e.g., ‘itching scabies’), (8) pregnancy (e.g., ‘scabies in pregnant women’), (9) information (e.g., ‘scabies Robert-Koch-Institute’), (10) surroundings (e.g., ‘scabies upholstered furniture’), (11) public institutions (e.g., ‘scabies in school’), (12) translation (e.g., ‘scabies in Persian’), (13) differential diagnosis (e.g., ‘atopic dermatitis or scabies’), and (14) diagnostics (e.g., ‘rapid test scabies’). Keywords that did not fit into any of these categories were assigned to the category (15) miscellaneous (e.g., ‘is scabies fatal’). Each search term was assigned to the category in which it fitted best.

### Statistical analysis

Descriptive data were generated for all variables. To compare the search volume of the cities and federal states, the search volume was set in relation to the respective number of inhabitants and then expressed as search volume per 100,000 inhabitants [20, 21]. To assess seasonal trends and differences in interest within the selected regions, one-way analysis of variance (ANOVA) was used after checking that the assumptions were fulfilled. Bonferroni test was used for post-hoc analysis. In addition, it was assessed whether there was a correlation between the average number of search queries and the population density in the federal sates. For all analysis, IBM SPSS Statistics Version 26 (IBM Corporation, Armonk, NY, USA) was used. For Fig. 3, Geodata from the German Federal Agency for Cartography and Geodesy [22] were used to determine administrative boundaries using a geographic information system, QGIS, version 2.14.22 (QGIS Development Team) [23].

## Results

Overall, 572 keywords related to scabies were identified by the Google Ads Keyword Planner (Online Resource 1), which generated a search volume of 11,414,180 searches between January 2016 and December 2019 across Germany as a whole. Accordingly, the nationwide search volume per 100,000 inhabitants was 13,727, resulting in 3432 searches per year on average. The five most frequently searched terms per 100,000 inhabitants were the German lay word for scabies (‘krätze’, *n* = 10,316), scabies therapy (*n* = 384), scabies (*n* = 382), scabies transmission (*n* = 172), and scabies home remedies (*n* = 155).

### Course of time

**Fig. 1:**
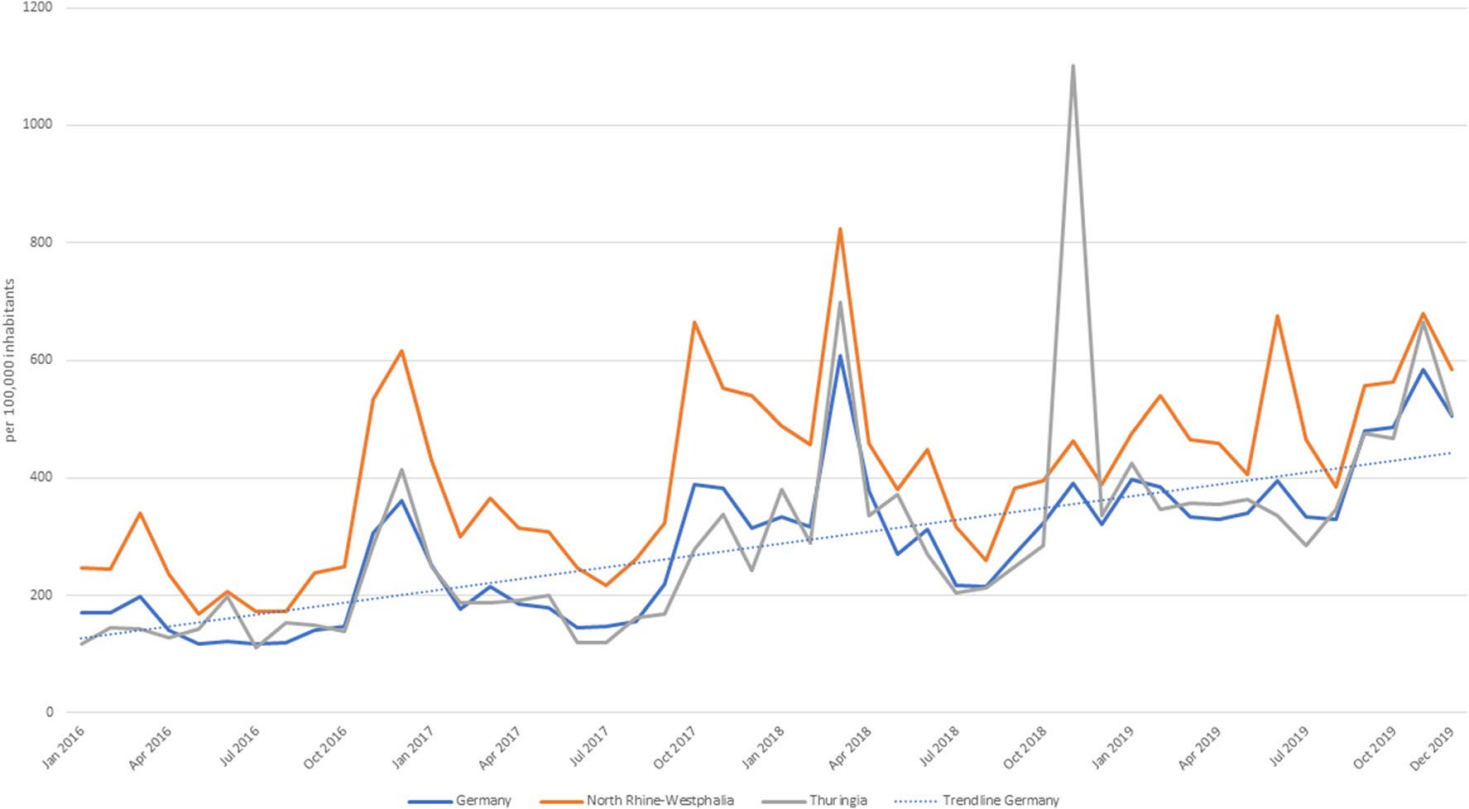
Trends in Google search volume per 100,000 inhabitants for scabies-related keywords in Germany as a whole and selected federal states from January 2016 to December 2019

**Fig. 2:**
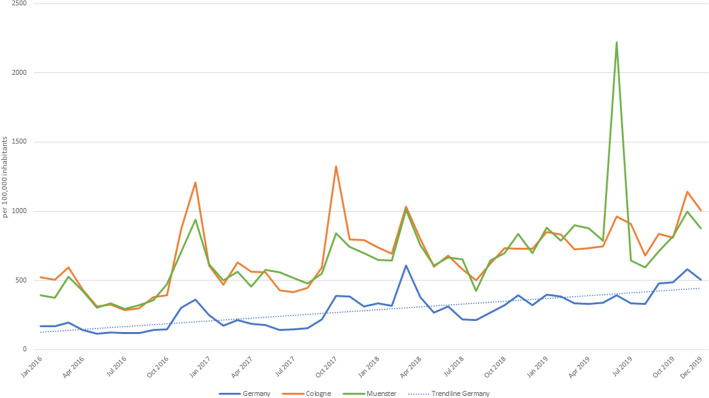
Trends in Google search volume per 100,000 inhabitants for scabies-related keywords in Germany as a whole and selected cities from January 2016 to December 2019

In general, the search volume was higher in the winter months (October–March) than in the summer months (April to September, *p* = 0.004, Fig. 1). The search volume per 100,000 inhabitants was higher in 2019 (average searches ± SD: 4898.3 ± 408.2) and 2018 (average searches ± SD: 3956.6 ± 329.7) than in 2017 (average searches ± SD: 2759.7 ± 230.0, 2019: *p* < 0.001; 2018: *p* = 0.053) and 2016 (average searches ± SD: 2112.4 ± 176.0, 2019: *p* < 0.001; 2018: *p* = 0.001). Accordingly, the number of search queries increased 2.3-fold from 2016 to 2019 (Online Resource 2). On the national level, most searches were recorded in March 2018 (*n* = 608 searches/100,000 inhabitants). On a state level, a peak in Thuringia was observed in November 2018 (*n* = 1101 searches/100,000 inhabitants, Fig. 1) apart from the peak in March 2018. On a city level, in Cologne for example, the number of searches was particularly high in December 2016 (*n* = 1208 searches/100,000 inhabitants) and October 2017 (*n* = 1325 searches/100,000 inhabitants). In June 2019, there was a peak in Muenster (*n* = 2221 searches/100,000 inhabitants, Fig. 2).

### Regional differences

**Fig. 3:**
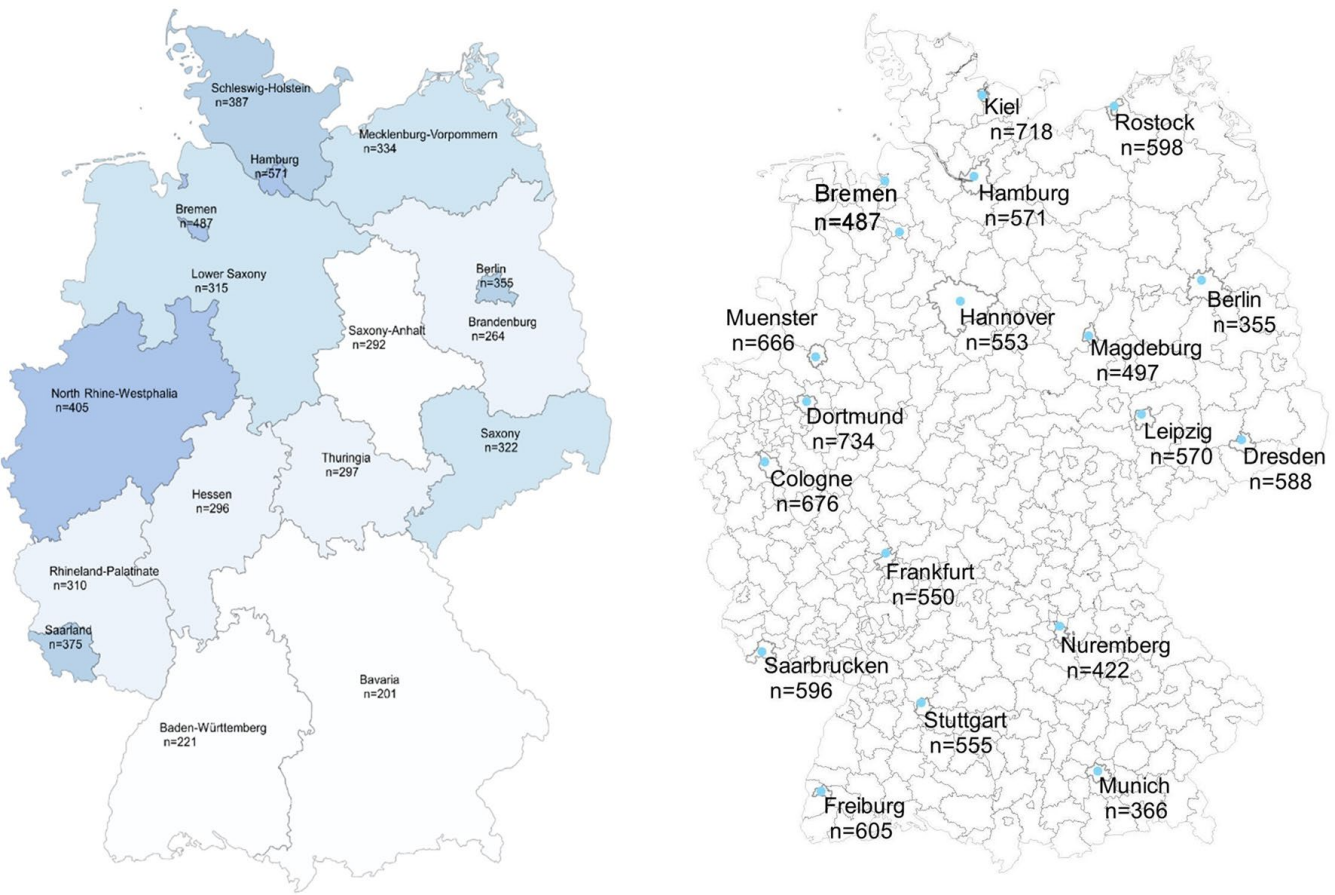
Number of search queries per 100,000 inhabitants for scabies-related keywords in the 13 German federal states, 15 German cities, and 3 city-states from January 2016 to December 2019

The nationwide average number of searches per month was 285.9 (± SD 125.0) searches/100,000 inhabitants. Among the federal states, Hamburg (*n* = 570.8 (± SD 231.0) searches/100,000 inhabitants) and North Rhine-Westphalia (*n* = 405.5 (± SD 153.8) searches/100,000 inhabitants) had the highest average monthly search volume, whereas Bavaria had the fewest (*n* = 201.0 (± SD 91.0) searches/100,000 inhabitants, Fig. 3). Accordingly, a positive correlation between the average searches and the population density was observed (*r* = 0.527, *p* = 0.036). Among the cities examined, Dortmund (*n* = 734.1 (± SD 254.1) searches/100,000 inhabitants) and Kiel (*n* = 717.5 (± SD 256.8) searches/100,000 inhabitants) had the highest search volume per month, whereas the lowest number of searches was recorded in Munich (*n* = 366.1 (± SD 123.3) searches/100,000 inhabitants, Fig. 3).

### Categorization

Online Resource 3 shows the five most common categories according to the number of searches in Germany and each federal state. It was observed that the categories ‘general’, ‘therapy’, ‘localization’, and ‘way of infection’ were always the four most common categories. The proportion of searches which were assigned to ‘general’, for example, ranged between 82.2% across the whole of Germany and 59.3% in Bremen. Except for Berlin, Bremen, Hamburg, Rhineland-Palatinate, Saarland, and Schleswig–Holstein, people more frequently searched for scabies in children than in animals.

### Therapy

On a national level, 8.6% (*n* = 978,420) of the searches were related to scabies therapy. In general, 56.8% (*n* = 556,060) of the searches in Germany were unspecific regarding treatment and 30.6% (*n* = 299,650) of searches were related to home remedies for scabies. The German phrase for “over the counter” was contained in 2.6% (*n* = 25,920) of the keywords and 1.3% (*n* = 12,850) of searches were for natural remedies. Regarding the specific search queries for certain home remedies, most people searched for coconut oil (3.6%), lavender oil (2.6%), and baby oil (0.9%). In Magdeburg, around 18.5% (*n* = 4416 searches/100,000 inhabitants) of the searches focused on therapy, whereas in Berlin only 11.6% (*n* = 1987 searches/100,000 inhabitants) did. The analysis showed that there was, for example, a difference between Munich (*n* = 2299, *p* = 0.017) and Kiel (*n* = 5793, *p* = 0.017) regarding the number of searches/100,000 inhabitants (Online Resources 3 and 4).

### Localization

**Fig. 4:**
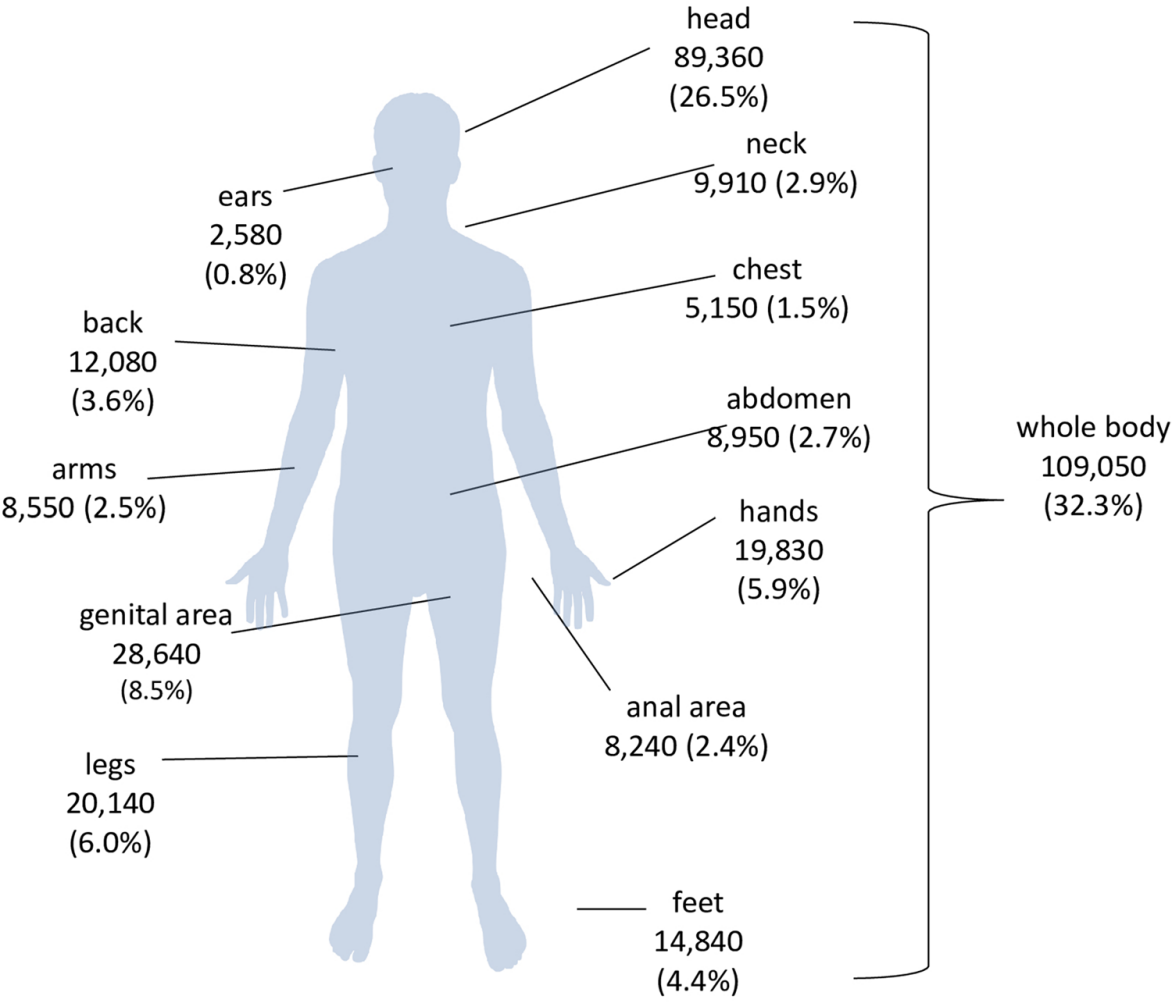
Absolute frequency of scabies-related Google searches in Germany from January 2016 to December 2019 grouped into body localizations

Nationwide, the most common search term regarding the localization of scabies was unspecific on the skin or on the body (*n* = 109,050, 32.3%). Head was the second most searched localization (*n* = 89,360, 26.5%), followed by the genital area (*n* = 28,640, 8.5%, Fig. 4).

In Bavaria, the highest proportion of searches were related to the whole body (36.2%), whereas Mecklenburg-Vorpommern had the lowest proportion (24.7%). Only in the federal states North Rhine-Westphalia (28.0%), Schleswig–Holstein (25.6%), and Hamburg (25.4%) as well as in the city Rostock (26.1%) the proportion of searches focused on the head was higher than that of searches related to the whole body (Online Resource 5).

## Discussion

The aim of the study was to assess the Google search behavior and search frequency related to scabies in Germany. It was observed that the number of search queries considerably increased between January 2016 and December 2019, that many people used more general search terms when searching for scabies, and that there was a high interest in home remedies for scabies treatment.

Overall, 11.4 million searches related to scabies were conducted on Google across Germany. In a comparable time period, nearly 20 million search queries on skin cancer [12] and 13.7 million search queries on pruritus were made in Germany via Google [11]. In the German general population, the prevalence of cutaneous melanoma is 0.12% and of non-melanocytic skin cancer is 0.65% [24], whereas chronic pruritus has a prevalence of 15.4% [25]. Although the prevalence of scabies might be considerably lower than that of pruritus, the number of searches related to pruritus was only slightly higher. Considering skin cancer, this health issue is frequently addressed in the media, whereas scabies is comparatively rarely discussed in public. Therefore, the high number of scabies-related searches underlines the relevance of scabies for the German population.

The number of search queries for scabies in Germany increased 2.3-fold between 2016 and 2019. Besides a higher health-related search behavior or a higher media coverage, the increase in search queries is also associated by an increase in documented scabies cases. In the federal state of Schleswig–Holstein, for example, an increase in scabies cases of almost threefold (from 4500 to 13,400 cases) was recorded within the 4 years [26]. In the same period, an increase of almost threefold in Google search queries was observed in this study. Moreover, in the region of Hannover, the number of scabies cases has more than doubled from 2017 to the end of 2019, with 218 cases in 2017 and 514 cases in 2019[27]. During the same time period, the number of online searches also doubled, which could indicate a correlation between registered cases and Google searches like it was previously demonstrated for other diseases [28, 29]. An increase in search queries over the years was also found in comparable studies on pruritus [11] and skin cancer [12]. The growing interest in scabies may be explained by an increased awareness of the disease, increased reliance on Google as the source of health information and an increased prevalence and growing burden of scabies in Germany [7]. The increase in scabies cases can be partly explained by the increasing number of refugees, who come from countries in Africa or the Middle East, where scabies has a high prevalence [2]. While in 2004 there were 11 cases of infectious diseases in refugee shelters, there were 331 cases in the year 2014. Of those cases, 19% were affected by scabies, indicating that refugees are disproportionately affected [30].

The number of searches increased each year during the colder months from October to March. This finding supports previous study results, which showed an increase in the incidence of scabies in the winter months compared to the summer [31, 32]. A possible explanation for this might be that scabies mites die faster at higher temperatures [33]. Furthermore, the natural skin barrier is more susceptible to lesions due to low temperatures [34], which in turn leads to increased itching [35] and thus further increases the burden for those affected by scabies. In addition, it could also play a role that people spend more time together indoors in winter than in summer [36], so that the risk of scabies transmission is increased. The peaks in search frequency in some cities could be due to local outbreaks. Interestingly, there was a particularly high number of searches in March 2018 in Germany. One possible explanation for this is the increased reporting on scabies in the media. In March 2018, a German health insurance company published a press release titled “Scabies on the rise—60% more prescriptions for scabies medication” [9]. This news was published as an announcement by the German Press Agency and was quoted in texts by various media houses [37, 38].

The average number of search queries per 100,000 inhabitants was more than twice as high in the city-state Hamburg and North Rhine-Westphalia than that in Bavaria [21]. The analysis showed that there was a positive correlation between the number of searches and the population density. One explanation for this could be the fact that scabies can be transmitted more quickly in regions where people live closer together [39], thus increasing public interest in the topic. Moreover, rural populations are on average older than urban populations and they use the Internet less frequently [40].

Other studies have already shown that web-search data can be used to answer medical and epidemiological questions. For example, previous studies have already shown positive correlations between Google search data and coronary heart disease [20] and Google search volume and dengue fever [21]. The frequent number of search queries on the topic of homemade remedies for scabies, in particular, illustrates this relevance. Nearly one-third of the searches regarding scabies therapy referred to home remedies. In contrast, antiscabiosa were relatively rarely searched despite the increasing prescriptions for them [9]. This circumstance might indicate that affected people want to treat themselves first before consulting a physician. This situation may also be an indication of current deficiencies in the therapy of scabies, whereby even with the availability of classic antiscabiosa, individuals try alternative remedies like oils to reduce their symptoms. This fact underlines the need for evidence-based information on effective treatment options.

The frequent searches for the localization of the scabies in the genital area are consistent with previous study results indicating that the genital area is one of the main localizations of scabies [41, 42]. The common occurrence in the genital area and the associated social embarrassment may be another factor that prevents affected individuals from immediately seeking medical help. Searches for scabies on the head were the second most frequently searched localizations, although scabies infections of the head are uncommon [43]. One explanation for this discrepancy could be that these search queries refer to scabies in children, as children are more likely to have scabies infections of the head [44–46]. Head lice infestations can also be mistaken for scabies and this may be another possible explanation for the increased search volume for scabies localized on the head [47].

There are some study limitations. Google Ads Keywords Planner tool estimates the monthly search volume according to its own algorithm, which may lead to slight deviations from the actual search volume. Since the study is based on data from an online search engine, only people with Internet access and who use it are considered. Older people may also be underrepresented in this study, as Internet use is more common in younger people [18]. Unfortunately, it is not possible to draw exact conclusions about the demographic data of Internet users. Furthermore, the function of automatic completion of a search term in Google can lead to a distortion of the frequencies of individual keywords. In addition, it should be noted that the study probably underestimates the interest of scabies in older and poorer populations, as these people are less likely to have access to the Internet. This fact suggests that the interest in scabies is even higher than the analysis suggests. Another limitation is that only Google was used for data collection. Even though over 95% of Germans use Google as their search engine [14], there is a potential selection bias.

In summary, the study provides an overview of the search interest regarding scabies of people in Germany. The increasing number of search queries reflects the growing relevance of the disease both on a national and local level. The high number of searches for the scabies therapy with home remedies possibly reflects a high number of underreported scabies cases, as people seeking home remedies for a disease do not necessarily consult a physician first. This simultaneously underlines the importance of educating the population about scabies so that affected individuals can consult a doctor and receive appropriate treatment earlier. Since official prevalence data on scabies are not available in Germany, alternative approaches such as Google search engine analyses are even more important for highlighting the relevance of scabies in the German population and for identifying growing public health issues regarding to scabies.

## Supplementary Information

Below is the link to the electronic supplementary material.

Supplementary file1 (DOCX 1202 KB)
