# Supplementary material for: The national burden of scabies in Germany: a population-based approach using Internet search engine data
Source: Infection. 2022 Feb 8;50(4):915–23. doi: 10.1007/s15010-022-01763-5 (PMC9338126; doi:10.1007/s15010-022-01763-5)
Supplement: Supplementary file 1 — Supplementary file1 (DOCX 1202 KB) [file 15010_2022_1763_MOESM1_ESM.docx]

**Supporting Information:**

**Online Resource 1.** Complete list of relevant German keywords for the main terms "scabies" and "krätze" in the Google Ads Keyword Planner program for the years 2016-2019

1. krätze
2. skabies
3. krätze behandlung
4. krätze hausmittel
5. krätze übertragung
6. borkenkrätze
7. krätze kinder
8. krätze anfang
9. die krätze
10. hausmittel gegen krätze
11. krätze bei kindern
12. hautmilben
13. milben krätze
14. krätze salbe
15. krätze krankheit
16. krätze tiere
17. krätze haut
18. krätze im gesicht
19. milben haut
20. ist krätze ansteckend
21. krätze gesicht
22. scabies behandlung
23. scabies therapie
24. krätze schwangerschaft
25. skabies therapie
26. krätze behandlung hausmittel
27. krätze mensch
28. krankheit krätze
29. behandlung krätze
30. milben unter der haut
31. milben auf der haut
32. krätze baby
33. krätze kleinkind
34. krätze kopfhaut
35. krätze englisch
36. hautkrankheit krätze
37. krätze finger
38. krätze in der schwangerschaft
39. krätze therapie
40. krätze ansteckungsgefahr
41. krätze woher
42. krätze an den händen
43. krätze scabies
44. hausmittel krätze
45. krätze am fuß
46. krätze fuß
47. krätze nach behandlung
48. krätze rücken
49. selbstbehandlung krätze
50. krätze am kopf
51. krätze kleidung im schrank
52. scabies milben
53. rki krätze
54. scabies krätze
55. krätze am bauch
56. krätze am hals
57. krätzemittel
58. krätze am po
59. rki scabies
60. hautkrätze
61. krätze schule
62. krätzmilben behandlung
63. krätze bauch
64. krätze auf dem kopf
65. krätze juckreiz
66. hautmilben mensch
67. krätze im intimbereich
68. krätze am bein
69. krätze intimbereich
70. krätze oberschenkel
71. kokosöl gegen krätze
72. gegen krätze
73. krätze arm
74. krätze kleidung
75. scabies infektion
76. behandlung von krätze
77. krätze am anfang
78. krätze in der schule
79. krätze an den beinen
80. krätze am arm
81. chronische krätze
82. milben in der haut
83. krätze am rücken
84. krätze 2019
85. krätze hautkrankheit
86. krätze selbsttest
87. krätze ansteckungszeit
88. milben am körper
89. krätze natürlich behandeln
90. milbenspray krätze
91. rki skabies
92. ist krätze tödlich
93. krätze in der kita
94. hausmittel gegen milben auf der haut
95. robert koch institut krätze
96. krätze po polsku
97. haut krätze
98. krätze brust
99. krätze kopf
100. milben behandlung
101. krätzebefall
102. krätze am hoden
103. krätze rki
104. krätze oder neurodermitis
105. krätzmilbe haut
106. krätze 36
107. krätze wäsche
108. lavendelöl gegen krätze
109. katze krätze
110. krätze hals
111. kann krätze von alleine weggehen
112. krätze am ganzen körper
113. scabies haut
114. krätze krankenhaus
115. krätze bein
116. krätze tödlich
117. hausmittel bei krätze
118. scabies rki
119. krätze bei säuglingen
120. juckreiz krätze
121. krätze behandlung erfolgreich
122. krätzemittel rezeptfrei
123. skabies milben
124. krätze bei menschen
125. skabies rki
126. krätze bei schwangeren
127. ansteckungsgefahr krätze
128. krätze doccheck
129. krätze po
130. krätze behandlung rezeptfrei
131. milben haut mensch
132. schwefelseife krätze
133. krätze symptome kinder
134. krätze am körper
135. krätze hoden
136. baby krätze
137. desinfektionsmittel gegen krätze
138. therapie krätze
139. krätze kita
140. leichte krätze
141. behandlung bei krätze
142. neurodermitis oder krätze
143. krätze auf der haut
144. scabies anfang
145. scabies hausmittel
146. krätze bei erwachsenen
147. krätze desinfektionsmittel
148. krätze selbst behandeln
149. saubere krätze
150. krätzmilbe hausmittel
151. krätze scheide
152. behandlung scabies
153. krätze infektion
154. krätzmilben mensch
155. milbeninfektion
156. krätze nach der behandlung
157. krätze auf tiere übertragbar
158. krätze auf englisch
159. krätze rezeptfrei
160. norwegische krätze
161. krätze erwachsene
162. krätze nacken
163. krätze am finger
164. krätze auf hunde übertragbar
165. anfang krätze
166. habe ich krätze
167. krätze salbe permethrin
168. nach krätze behandlung
169. krätze woher kommt das
170. anfang von krätze
171. krätze beim menschen
172. krätze bei kindern hausmittel
173. scabies kopfhaut
174. krätze auf der kopfhaut
175. lavendelöl krätze
176. skabizide
177. nesselsucht oder krätze
178. krätze bei tieren
179. krätze oder allergie
180. milben mensch haut
181. krätze allergie
182. ist die krätze ansteckend
183. therapie scabies
184. hautmilben bei menschen
185. scabies tiere
186. ansteckungszeit krätze
187. krätze durch katze übertragen
188. scabies gesicht
189. krätze unter der haut
190. hilft desinfektionsmittel gegen krätze
191. krätze und schwangerschaft
192. skabies infektion
193. krätze am oberschenkel
194. naturasil krätze
195. krusten krätze
196. krätze wunden
197. krätze am glied
198. hausmittel gegen scabies
199. parasitäre krätze
200. scabies schwangerschaft
201. hab ich krätze
202. ist krätze auf tiere übertragbar
203. scabies intimbereich
204. skabies bei kindern
205. krätze woher kommt sie
206. die krankheit krätze
207. krätze in kita
208. milben scabies
209. kann krätze auf tiere übertragen werden
210. naturasil gegen krätze
211. krätze kinder symptome
212. krätze robert koch institut
213. krätze an der scheide
214. kokosöl krätze
215. die krätze symptome
216. krätze symptome nach behandlung
217. milbensalbe
218. krätze ohne behandlung
219. krätze schnelltest
220. krätze neurodermitis
221. krätze an beinen
222. krätze knie
223. scabies krankheit
224. hausmittel gegen krätzmilben
225. krätze an der brust
226. milbenspray gegen krätze
227. envira milbenspray krätze
228. krätze wohnung
229. krätzmilben woher
230. krätze ohr
231. krätze intimbereich behandlung
232. krätze am auge
233. krätze behandlung kinder
234. krätze an den fingern
235. krätze in schwangerschaft
236. krätze vorbeugung
237. krätze an den armen
238. nesselsucht nach krätze behandlung
239. behandlung gegen krätze
240. versteckte krätze
241. krätze auf kopfhaut
242. milben unter der haut mensch
243. krätze alternativ behandeln
244. scabies im gesicht
245. scabies orale therapie
246. krätze richtig behandeln
247. borkenkrätze symptome
248. krätze unter der brust
249. krätze leicht
250. ansteckungsgefahr bei krätze
251. krätze kokosöl
252. scabies mensch
253. scabies kleinkind
254. babyöl gegen krätze
255. krätze gegenmittel
256. milben auf haut
257. krätze homöopathisch behandeln
258. krätze nicht behandeln
259. neurodermitis krätze
260. skabies doccheck
261. krätze verschwindet nicht
262. krätze nur an den beinen
263. krätze oder nesselsucht
264. verschwindet krätze von alleine
265. krätze durch katze
266. krätze hunde übertragbar
267. nelkenöl gegen krätze
268. krätze hausmittel behandeln
269. krätze beim kind
270. kann krätze auf hunde übertragen werden
271. krätze am ohr
272. krätzmilben kopfhaut
273. bullöse krätze
274. krätze durch hund
275. nach krätze behandlung juckreiz
276. ist die krätze tödlich
277. krätzmilben katze
278. granulomatöse krätze
279. krätze möbel behandeln
280. krätze bei neugeborenen
281. rezeptfreie krätzemittel
282. advantan milch krätze
283. krätze trockner
284. krätze im gesicht bei kindern
285. krätze salbe infectoscab
286. milben intimbereich
287. schwangerschaft krätze
288. krätze an den hoden
289. desinfektionsmittel krätze
290. krätze apotheken umschau
291. krätze im nacken
292. krätze baby krankenhaus
293. krätze augenlid
294. ist krätze auf hunde übertragbar
295. krätze badezusatz
296. mit krätze in die schule
297. krätze sofa
298. neurodermitis und krätze
299. schwefelsalbe krätze
300. milben therapie
301. scabies nach behandlung
302. krätze hund mensch
303. krätze von mensch auf hund
304. krätze lavendelöl
305. krätze auf katzen übertragbar
306. hausmittel gegen krätze milben
307. krätze kinder behandlung
308. krätze naturheilmittel
309. behandlung von krätze beim menschen
310. krätze polstermöbel
311. krätze neugeborene
312. krätze in schule
313. milben mensch behandlung
314. desinfektionsmittel bei krätze
315. krätze bei frauen
316. krätze sex
317. krätze behandlung im krankenhaus
318. scabies katze
319. therapie bei krätze
320. krätze im ohr
321. krätze auf dem rücken
322. krätze möbel
323. krätze an füßen
324. bzga krätze
325. borkenkrätze behandlung
326. kann krätze von alleine heilen
327. krätze kind gesicht
328. krätze nase
329. hautkrankheit scabies
330. milbenspray bei krätze
331. scabies in der schwangerschaft
332. krätze behandlung salbe
333. naturheilmittel gegen krätze
334. krätze in den haaren
335. therapie skabies
336. krätze heilmittel
337. krätze nebenwirkungen
338. lavendelöl bei krätze
339. nach krätze behandlung starker juckreiz
340. krätze bett
341. dampfreiniger gegen krätze
342. krätzmilben intimbereich
343. kopfhaut krätze
344. krätze nesselsucht
345. juckreiz bei krätze
346. kokosöl bei krätze
347. krätze youtube
348. krätze ist das ansteckend
349. kind hat krätze
350. schwefelsalbe gegen krätze
351. badezusatz gegen krätze
352. krätze netdoktor
353. krätze übertragbar auf tiere
354. krätzmilben desinfektionsmittel
355. krätze übertragbar auf hunde
356. krankheit die krätze
357. nelkenöl krätze
358. krätzmilben im bett
359. rezeptfrei krätze
360. krätze zuhause behandeln
361. krätze intimbereich frau
362. apotheken umschau krätze
363. robert koch institut scabies
364. krätze am sack
365. krätzmilbe therapie
366. symptome krätze kinder
367. kammerjäger krätze
368. ist krätze
369. allergie oder krätze
370. krätze am after
371. krätze kopfhaut behandeln
372. milben im intimbereich
373. krätze im krankenhaus
374. krätze milben im bett
375. nodöse krätze
376. sind krätze ansteckend
377. heilmittel gegen krätze
378. krätze behandlung krankenhaus
379. krätze von mensch auf hund übertragbar
380. krätze auf persisch
381. krätze auf rumänisch
382. crotamiton krätze
383. krätze bei neurodermitis
384. krätze im krankenhaus behandeln
385. krätze behandlung wohnung
386. krätze dampfreiniger
387. kammerjäger bei krätze
388. krätze nach behandlung ansteckend
389. krätze am kopf behandeln
390. krätzmilben kleidung
391. nesselsucht nach krätze
392. rezeptfrei gegen krätze
393. krätze kammerjäger
394. naturasil scabies kaufen
395. krätze im auge
396. krätze schnell behandeln
397. krätze fingernägel
398. krätze und neurodermitis
399. nesselsucht krätze
400. nach der krätze behandlung
401. reinfektion krätze
402. krätze behandlung kleidung
403. krätze nicht ansteckend
404. hautkrankheit milben
405. scabies desinfektionsmittel
406. krätze im intimbereich behandeln
407. neurodermitis nach krätze
408. krätze auf dem kopf behandeln
409. krätze bei kindern behandeln
410. milbopax krätze
411. krätze ansteckend für hunde
412. krätze offene wunden
413. krätze naturheilkunde
414. krätze behandlung permethrin
415. crotamiton rezeptfrei
416. krankheit scabies
417. krätze auf der brust
418. krätze welche salbe
419. vogel krätze
420. krätze unter den füßen
421. krätze mit hausmittel behandeln
422. schnelltest krätze
423. krätze erfolgreich behandeln
424. krätze mit desinfektionsmittel behandeln
425. krätze übertragung kleidung
426. krätze selbst heilen
427. anti krätze
428. krätze behandlung juckreiz
429. milben krätze behandlung
430. youtube krätze
431. parasiten krätze
432. krätze bettdecke
433. milbopax gegen krätze
434. krätze im sommer
435. krätze symptome bei kindern
436. tote krätzmilben
437. nach behandlung von krätze
438. starker krätzebefall
439. permethrin salbe gegen krätze
440. ist krätze auf katzen übertragbar
441. ich krieg die krätze
442. krätze desinfektionsspray
443. milbeninfektion haut
444. krätze in der familie
445. krätze behandlung nicht erfolgreich
446. zinksalbe gegen krätze
447. krätze mensch hund
448. scabies natürlich behandeln
449. krätze an der kopfhaut
450. hautpflege nach krätze
451. krätze salbe anwendung
452. krätze in der nase
453. krätze effektiv behandeln
454. nach der behandlung von krätze
455. krätze umgebungsspray
456. krätze naturheilkundlich behandeln
457. krätze nur an den händen
458. krätze behandlung in der schwangerschaft
459. gegen scabies
460. haut nach krätze
461. milben unter haut
462. envira milbenspray gegen krätze
463. scabies woher
464. krätze durch unhygiene
465. krätze katzen ansteckend menschen
466. hilft milbenspray gegen krätze
467. krätze baby behandlung
468. krätze nur am arm
469. freund hat krätze
470. ansteckungszeit bei krätze
471. allergie krätze
472. krätzmilben unter der haut
473. krätze von neurodermitis unterscheiden
474. habe ich die krätze
475. krätze von mensch auf katze übertragbar
476. milbenbefall auf der haut
477. hausmittel scabies
478. krätze nichts hilft
479. krätze 2018
480. krätze teppich
481. krätze in englisch
482. krätze durch milben
483. krätze nur an beinen
484. milben in haut
485. netdoktor krätze
486. krätze von mensch auf katze
487. krätze bei kindern im gesicht
488. krätze von mensch auf tier
489. krätze wäsche behandeln
490. behandlung von scabies
491. krätze und katzen
492. skabizide kaufen
493. infektion krätze
494. crotamitex krätze
495. scabies rezeptfrei
496. ich habe krätze
497. krätze ansteckend für tiere
498. krankenhaus krätze
499. kann krätze von tieren übertragen werden
500. krätzmilben bei kindern
501. kann krätze von hunden übertragen werden
502. krätze behandlung ivermectin
503. krätze behandlung bettwäsche
504. krätze couch
505. krätze übertragbarkeit
506. gegen krätze hausmittel
507. krätze vom hund auf mensch
508. krätze schwangerschaft behandlung
509. milben in der haut beim menschen
510. envira krätze
511. krätze meerschweinchen
512. resistente krätze
513. krätze krankenhaus behandeln
514. krätze behandlung bei säuglingen
515. nesselsucht durch krätze
516. kita krätze
517. krätze über kleidung übertragbar
518. hausmittel gegen hautmilben
519. krätze scabies behandlung
520. scabies schule
521. hautmilben beim menschen
522. desinfektionsspray gegen krätze
523. norwegische scabies
524. krätze nur am rücken
525. gürtelrose oder krätze
526. partner hat krätze
527. milben haut hausmittel
528. milbenbefall mensch behandlung
529. hab ich die krätze
530. krätze vorbeugende behandlung
531. erste symptome bei krätze
532. infectopharm krätze
533. borkenkrätze kopfhaut
534. ich glaube ich habe krätze
535. krätze in der schwangerschaft behandeln
536. haut krätze ansteckend
537. läusemittel gegen krätze
538. krätze milben behandlung
539. krätze behandlung wäsche
540. krätze in der wohnung
541. krätzmilben bei katzen
542. krätze milben kleidung
543. nach krätze
544. milbeninfektion mensch
545. krätze bei kindern woher
546. ist krätze ansteckend für hunde
547. schädlingsbekämpfung krätze
548. desinfektionsmittel gegen krätzmilben
549. milben bei menschen übertragbar
550. scabies auf tiere übertragbar
551. krätze orale behandlung
552. krätze medikamente permethrin
553. naturheilmittel krätze
554. hunde krätze ansteckend
555. prophylaktische behandlung krätze
556. anti krätzemittel
557. krätzmilben ansteckungsgefahr
558. krätzmilben behandlung hausmittel
559. krätze am pennis
560. scabies nach der behandlung
561. hautmilben hausmittel
562. krätze wäsche desinfizieren
563. krätze woher kommen die
564. krätze nach eincremen
565. milben krätze übertragung
566. krätze kleidung luftdicht
567. krätzmilben nach behandlung
568. krätze katze behandlung
569. medizinisch krätze
570. krätzmilbe natürlich behandeln
571. scabies im intimbereich
572. schmutz krätze

**Online Resource 2.** Nationwide search volume in the summer months (April to September) and in the winter months (October to March) from January 2016 to December 2019

| **Period of time** | **Search queries in Germany** |
| --- | --- |
| April to September 2016 | 631,150 |
| January to March and October to December 2016 | 1,125,390 |
| April to September 2017 | 857,840 |
| January to March and October to December 2017 | 1,436,880 |
| April to September 2018 | 1,381,810 |
| January to March and October to December 2018 | 1,908,180 |
| April to September 2019 | 1,835,290 |
| January to March and October to December 2019 | 2,237,640 |

**Online Resource 3.** Number of searches related to scabies in the five most common categories in each federal state during the period from January 2016 to December 2019; data per 100,000 inhabitants

|  | **Germany** | **Baden-Wuerttemberg** | **Bavaria** | **Berlin** | **Brandenburg** |
| --- | --- | --- | --- | --- | --- |
| 1 | General (n=11,289, 82.2%) | General (n=8,306, 78.2%) | General (n=7,607, 78.8%) | General (n=12,482, 73.2%) | General (n=8,574, 67.8%) |
| 2 | Therapy  (n=1,177, 8.6%) | Therapy  (n=1,063, 10.0%) | Therapy  (n=935, 9.7%) | Therapy  (n=1,987, 11.6%) | Therapy  (n=1,667, 13.2%) |
| 3 | Localization  (n=406, 3.0%) | Localization  (n=423, 4.0%) | Localization  (n=379, 3.9%) | Localization  (n=843, 4.9%) | Localization  (n=797, 6.3%) |
| 4 | Way of infection  (n=272, 2.0%) | Way of infection  (n=208, 20%) | Way of infection  (n=185, 1.9%) | Way of infection  (n=386, 2.3%) | Way of infection  (n=308, 2.4%) |
| 5 | Children (n=168, 1.2%) | Children (n=126, 1.2%) | Children (n=111, 1.1%) | Animals (n=233, 1.4%) | Children(n=300, 2.4%) |
|  | All categories (n=13,727) | All categories (n=10,624) | All categories (n=9,648) | All categories (n=17,060) | All categories (n=12,649) |

|  | **Bremen** | **Hamburg** | **Hessen** | **Mecklenburg-Vorpommern** |
| --- | --- | --- | --- | --- |
| 1 | General (n=13,869, 59.3%) | General (n=19,357, 70.7%) | General (n=10,828, 76.2%) | General (n=10,583, 66.0%) |
| 2 | Therapy (n=3,739, 16.0%) | Therapy (n=3,412, 12.5%) | Therapy (n=1,486, 10.5%) | Therapy (n=2,215, 13.8%) |
| 3 | Localization (n=2,064, 8.8%) | Localization (n=1,496, 5.5%) | Localization (n=625, 4.4%) | Localization (n=1,096, 6.8%) |
| 4 | Way of infection (n=723, 3.1%) | Way of infection  (n=676, 2.5%) | Way of infection (n=295, 2.1%) | Way of infection (n=426,2.7%) |
| 5 | Animals (n=524, 2.2%) | Animals (n=427, 1.6%) | Children (n=206, 1.4%) | Children (n=326, 2.0%) |
|  | All categories (n=23,384) | All categories (n=27,393) | All categories (n=14,212) | All categories (n=16,043) |

|  | **Lower Saxony** | **North Rhine-Westphalia** | **Rhineland-Palatinate** | **Saarland** |
| --- | --- | --- | --- | --- |
| 1 | General (n=11,593, 76.8%) | General (n=15,620, 80.3%) | General (n=10,943, 73.6%) | General (n=11,598, 64.4%) |
| 2 | Therapy (n=1,611, 10.7%) | Therapy (n=1,810, 9.3%) | Therapy (n=1,691, 11.4%) | Therapy (n=2,454, 13.6%) |
| 3 | Localization (n=593, 3.9%) | Localization (n=626,3.2%) | Localization (n=742, 5.0%) | Localization (1,436 = 8.0%) |
| 4 | Way of infection (n=360, 2.4%) | Way of infection (n=447, 2.3%) | Way of infection (n=329, 2.2%) | Way of infection n (499 = 2.8%) |
| 5 | Children (n=185, 1.2%) | Children (n=219, 1.1%) | Animals (n=215, 1.4%) | Animals (n=338, 1.9%) |
|  | All categories (n=15,096) | All categories (n=19,463) | All categories (n=14,869) | All categories (n=18,002) |

|  | **Saxony** | **Saxony-Anhalt** | **Schleswig-Holstein** | **Thuringia** |
| --- | --- | --- | --- | --- |
| 1 | General (n=11,549, 74.7%) | General (n=9,562, 68.2%) | General (n=13,029, 70.1%) | General (n=10,014, 70.3%) |
| 2 | Therapy (n=1,734, 11.2%) | Therapy (n=1,848, 13.2%) | Therapy (n=2,353,12.7%) | Therapy (n=1,739, 12.2%) |
| 3 | Localization (n=698, 4.5%) | Localization (n=859, 6.1%) | Localization (n=1,008, 5.4%) | Localization (n=865, 6.1%) |
| 4 | Way of infection (n=322, 2.1%) | Way of infection (n=337, 2.4%) | Way of infection (n=505, 2.7%) | Way of infection (n=318, 2.2%) |
| 5 | Children (n=237, 1.5%) | Children (n=309, 2.2%) | Animals (n=310, 1.7%) | Children (n=243, 1.7%) |
|  | All categories (n=15,452) | All categories (n=14,022) | All categories (n=18,598) | All categories (n=14,247) |

**Online Resource 4.** Number of searches related to scabies in the three most common categories in 15 cities during the period from January 2016 to December 2019; data per 100,000 inhabitants

|  |  | **Dortmund** | **Dresden** | **Frankfurt** | **Freiburg** | **Hannover** |
| --- | --- | --- | --- | --- | --- | --- |
| 1 | General | n=22,347, 63.4% | n=18,440, 65.4% | n=17,158, 65.0% | n=16,509, 56.9% | n=16,117, 60.7% |
| 2 | Therapy | n=4,979, 14.1% | n=3,842, 13.6% | n=3,690, 14.0% | n=4,934, 17.0% | n=4,003, 15.1% |
| 3 | Localization | n=2,753, 7.8% | n=2,066, 7.3% | n=2,069, 7.8%) | n=2,788, 9.6% | n=2,273, 8.6% |

|  |  | **Kiel** | **Cologne** | **Leipzig** | **Magdeburg** | **Munich** |
| --- | --- | --- | --- | --- | --- | --- |
| 1 | General | n=18,861, 54.8% | n=22,000, 67.8% | n=17,076, 62.4% | n=13,058, 54.8% | n=11,935, 67.9% |
| 2 | Therapy | n=5,793, 16.8% | n=4,155, 12.8% | n=4,096, 15.0% | n=4,416, 18.5% | n=2,299, 13.1% |
| 3 | Localization | n=3,559, 10.3% | n=2,112, 6.5% | n=2,256, 8.2% | n=2,354, 9.9% | n=1,257, 7.2% |

|  |  | **Muenster** | **Nuremberg** | **Rostock** | **Saarbrucken** | **Stuttgart** |
| --- | --- | --- | --- | --- | --- | --- |
| 1 | General | n=18,462, 57.8% | n=12,138, 59.9% | n=15,836, 55.1% | n=15,984, 55.9% | n=17,260, 64.7% |
| 2 | Therapy | n=5,036, 15.8% | n=3,197, 15.8% | n=4,878, 17.0% | n=4,692, 16.4% | n=3,659, 13.7% |
| 3 | Localization | n=2,844, 8.9% | n=1,896, 9.4% | n=2,810, 9.8% | n=2,816, 9.8% | n=2,202, 8.3% |

**Online Resource 5.** Number of searches related to scabies in the five most common body localizations in each federal state and the city of Rostock during the period from January 2016 to December 2019; data per 100,000 inhabitants

|  | **Localization** | **Baden-Wuerttemberg** | **Germany** | **Bavaria** | **Berlin** | **Brandenburg** |
| --- | --- | --- | --- | --- | --- | --- |
| 1 | whole body | n=148, 35,0% | n=131, 32.3% | n=137, 36.2% | n=232, 27.5% | n=198, 24.9% |
| 2 | head | n=103, 24.4% | n=107, 26.5% | n=93, 24,6% | n=214, 25.4% | n=186, 23.4% |
| 3 | genital area | n=43, 10.2% | n=34, 8.5% | n=36, 9.5% | n=89, 10.5% | n=92, 11.6% |
| 4 | legs | n=26, 6.2% | n=24, 6,0% | n=24, 6.4% | n=65, 7.7% | n=68, 8.6% |
| 5 | hands | n=20, 4.6% | n=24, 5,9% | n=17, 4.4% | n=45, 5.4% | n=45, 5.7% |

|  | **Localization** | **Bremen** | **Hamburg** | **Hessen** | **Mecklenburg-Vorpommern** |
| --- | --- | --- | --- | --- | --- |
| 1 | whole body | n=515, 25,0% | n=363, 24.3% | n=192, 30.6% | n=270, 24.7% |
| 2 | head | n=463, 22,4% | n=380, 25,4% | n=155, 24.9% | n=248, 22.6% |
| 3 | genital area | n=240, 11.6% | n=182, 12.2% | n=67, 10.8% | n=111, 10.1% |
| 4 | legs | n=182, 8.8% | n=117, 7.8% | n=43, 6.9% | n=107, 9.7% |
| 5 | hands | n=120, 5.8% | n=84, 5.6% | n=30, 4.9% | n=70, 6.3% |

|  | **Localization** | **Lower Saxony** | **North Rhine-Westphalia** | **Rhineland-Palatinate** | **Saarland** |
| --- | --- | --- | --- | --- | --- |
| 1 | whole body | n=160, 27.0% | n=172, 27.5% | n=207, 27.9% | n=383, 26.6% |
| 2 | head | n=155, 26.2% | n=175, 28.0% | n=177, 23.9% | n=341, 23.8% |
| 3 | genital area | n=63, 10.7% | n=60, 9.5% | n=83, 11.2% | n=166, 11.5% |
| 4 | legs | n=43, 7.2% | n=41, 6.5% | n=61, 8.3% | n=116, 8.1% |
| 5 | hands | n=34, 5.7% | n=40, 6.4% | n=39, 5.2% | n=77, 5.3% |

|  | **Localization** | **Saxony** | **Saxony-Anhalt** | **Schleswig-Holstein** | **Thuringia** | **Rostock** |
| --- | --- | --- | --- | --- | --- | --- |
| 1 | whole body | n=181, 25.9% | n=228, 26.6% | n=237, 23.5% | n=239, 27.7% | n=694, 24.7% |
| 2 | head | n=169, 24.2% | n=193, 22.5% | n=258, 25.6% | n=207, 23.9% | n=732, 26.1% |
| 3 | genital area | n=76, 10.9% | n=92, 10.7% | n=116, 11.5% | n=89, 10.3% | n=220, 7.8% |
| 4 | legs | n=59, 8.4% | n=69, 8.1% | n=88, 8.8% | n=68, 7.9% | n=254=9.0% |
| 5 | hands | n=41, 5.8% | n=51, 6.0% | n=56, 5.5% | n=47, 5.4% | n=158, 5.6% |

**
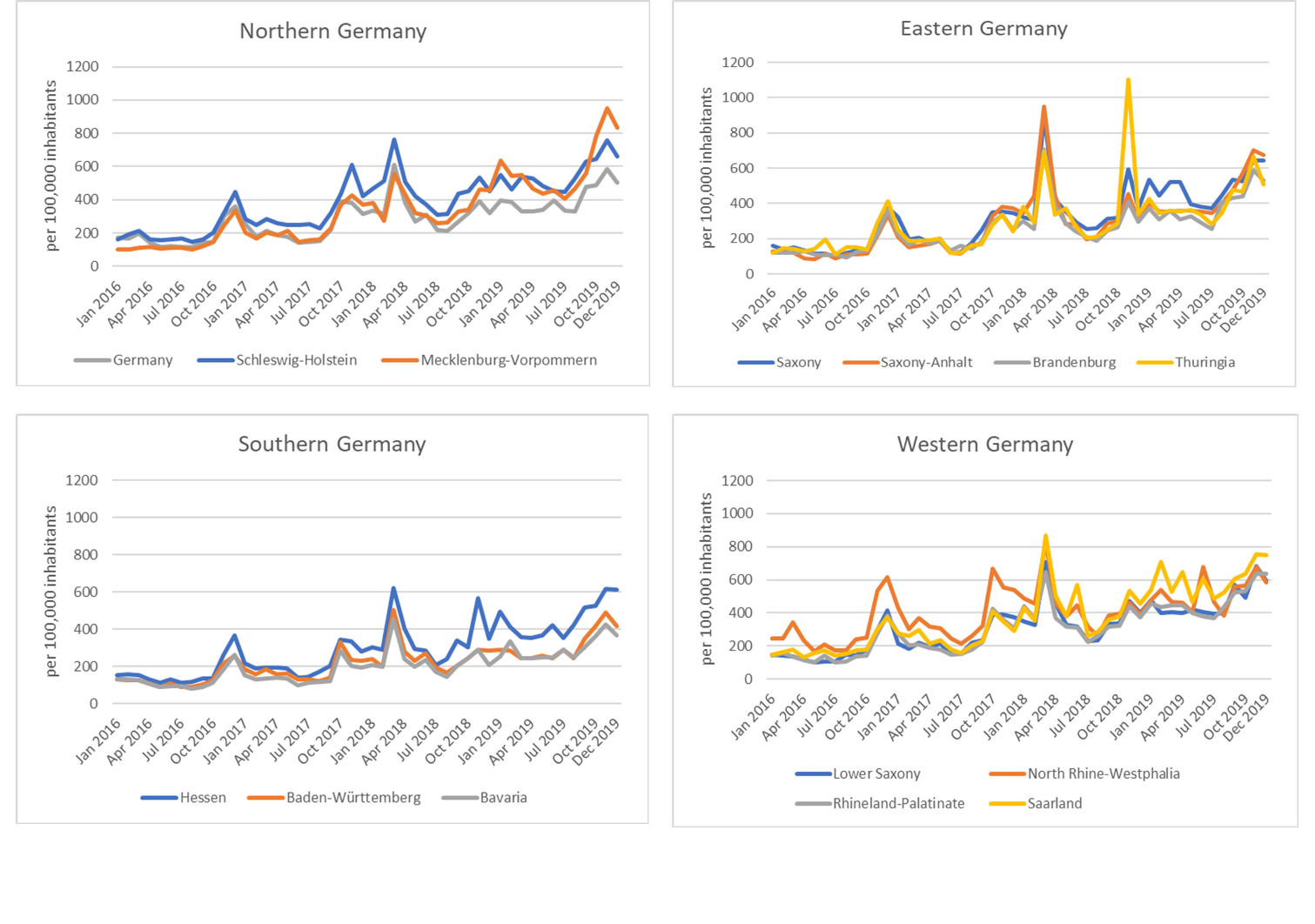
Online Resource 6.** Trends in Google search volume per 100,000 inhabitants for scabies-related keywords in Germany as a whole and its 13 federal states excluding the three city-states from January 2016 to December 2019

**
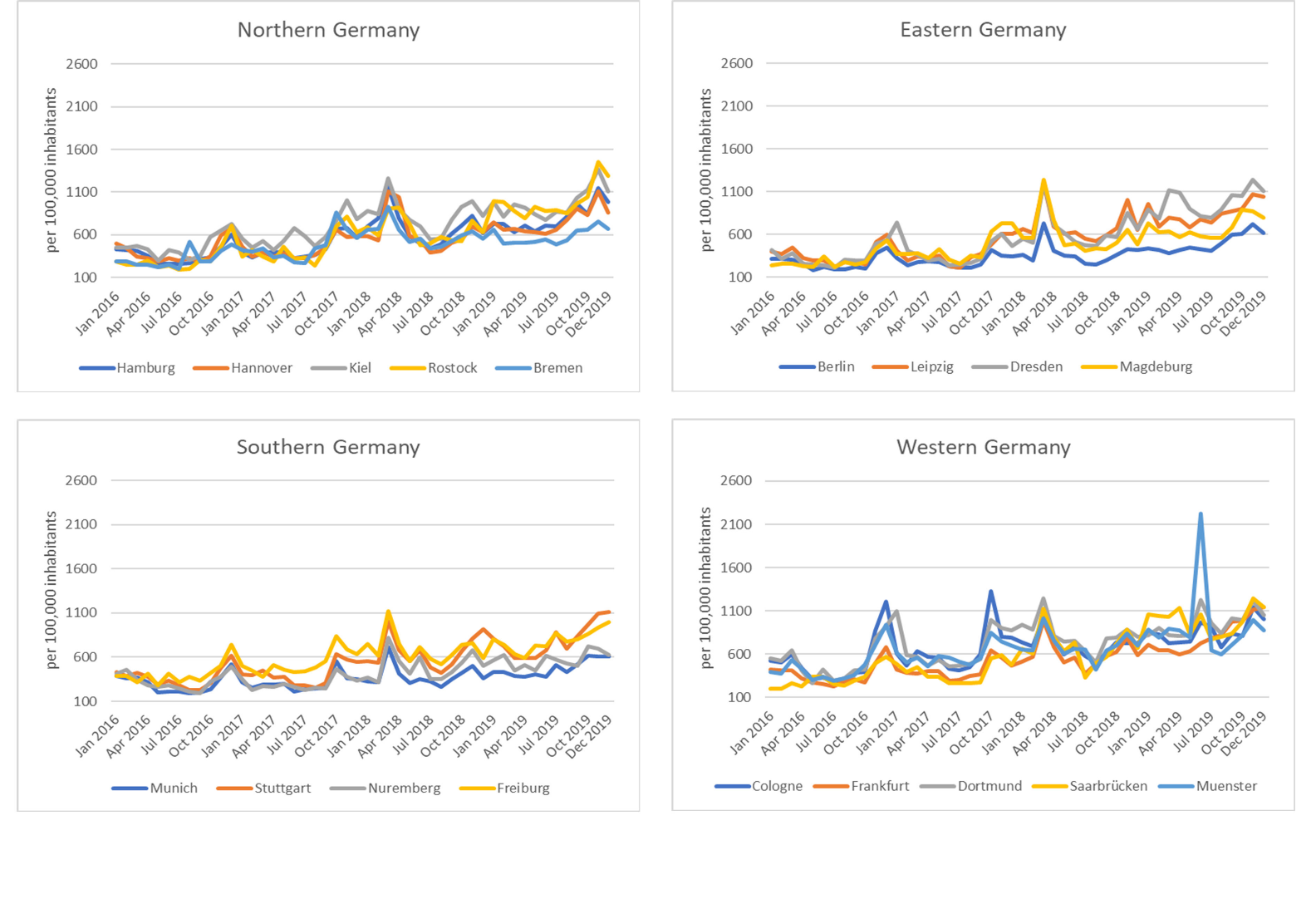
Online Resource 7.** Trends in number of Google searches per 100,000 inhabitants for scabies-related keywords in 15 German cities and 3 city-states from January 2016 to December 2019
